# Repeated epinephrine doses during prolonged cardiopulmonary resuscitation have limited effects on myocardial blood flow: a randomized porcine study

**DOI:** 10.1186/1471-2261-14-199

**Published:** 2014-12-20

**Authors:** Henrik Wagner, Michael Götberg, Bjarne Madsen Hardig, Malin Rundgren, Jonas Carlson, Matthias Götberg, David Zughaft, David Erlinge, Göran K Olivecrona

**Affiliations:** Department of Cardiology, Lund University, Lund, Sweden; Physio-Control/Jolife AB, Ideon Science Park, Lund, Sweden; Department of Anesthesiology and Intensive Care, Lund University, Lund, Sweden

**Keywords:** Cardiac arrest, Mechanical chest compressions, Epinephrine

## Abstract

**Background:**

In current guidelines, prolonged cardiopulmonary resuscitation (CPR) mandates administration of repeated intravenous epinephrine (EPI) doses. This porcine study simulating a prolonged CPR-situation in the coronary catheterisation laboratory, explores the effect of EPI-administrations on coronary perfusion pressure (CPP), continuous coronary artery flow average peak velocity (APV) and amplitude spectrum area (AMSA).

**Methods:**

Thirty-six pigs were randomized 1:1:1 to EPI 0.02 mg/kg/dose, EPI 0.03 mg/kg/dose or saline (control) in an experimental cardiac arrest (CA) model. During 15 minutes of mechanical chest compressions, four EPI/saline-injections were administered, and the effect on CPP, APV and AMSA were recorded. Comparisons were performed between the control and the two EPI-groups and a combination of the two EPI-groups, EPI-all.

**Result:**

Compared to the control group, maximum peak of CPP (P_max_) after injection 1 and 2 was significantly increased in the EPI-all group (p = 0.022, p = 0.016), in EPI 0.02-group after injection 2 and 3 (p = 0.023, p = 0.027) and in EPI 0.03-group after injection 1 (p = 0.013). At P_max_, APV increased only after first injection in both the EPI-all and the EPI 0.03-group compared with the control group (p = 0.011, p = 0.018). There was no statistical difference of AMSA at any P_max_. Seven out of 12 animals (58%) in each EPI-group versus 10 out of 12 (83%) achieved spontaneous circulation after CA.

**Conclusion:**

In an experimental CA-CPR pig model repeated doses of intravenous EPI results in a significant increase in APV only after the first injection despite increments in CPP also during the following 2 injections indicating inappropriate changes in coronary vascular resistance during subsequent EPI administration.

## Background

Routine use of epinephrine (EPI) during cardiopulmonary resuscitation (CPR) was first described in the early 1960-ies [1] and has ever since been recommended in guidelines during advanced CPR. Current guidelines recommend administration of 1 mg of EPI given intravenously every 3 – 5 minute Class IIb (LOE A) [2, 3]. Several clinical studies and randomized trials have reported an increased frequency of return of spontaneous circulation (ROSC) in out-of-hospital cardiac arrest (OHCA) after various dosages of repeated EPI administrations [4–7]. However, the increased frequency of ROSC does not convey improvements in discharge from hospital in good neurological condition or long term survival [4, 7, 8].

Both experimental and clinical studies have shown a higher frequency of ROSC when the coronary perfusion pressure (CPP) can be brought to a level > 15 mmHg before defibrillation during CPR-treatment [9–13]. Both increments in CPP and myocardial creatine phosphate are associated with altered ventricular fibrillation (VF) wavelets that in turn increase the possibility of a successful CPR [14]. Amplitude spectrum area (AMSA) represents a quantitative combined measure of the electrical activity of VF wavelets, that seems to be a predictor of defibrillation success [15] and other experimental studies have shown a correlation between CPP, AMSA, and defibrillation success [13, 16].

A Doppler flow wire can be used to measure coronary blood flow in patients with a stable circulation [17–19], and experimentally a good correlation has been demonstrated between CPP and the average blood flow (APV) in a coronary vessel assessed by a Doppler flow wire during mechanical chest compressions (MCC) [20].

Previous experimental studies have evaluated the effects of repeated doses of EPI on CPP [21, 22], VF-amplitude [23] and myocardial blood flow [24] during CPR, but all these studies have been designed to reflect the OHCA situation. The aim of this study was to assess CPP, coronary artery APV reflecting myocardial pressure and perfusion in addition to AMSA (reflecting bioelectrical activity) during CA caused by VF in an experimental situation adopted to the catheterisation laboratory setting, which includes a shorter untreated VF time and prolonged need for CPR while performing PCI [25].

## Methods

The study conformed to the guide for the care and use of laboratory animals, US National Institute of Health (NIH Publication No. 85-23, revised 1996) and was approved by the Malmö/Lund Committee for Animal Experiment Ethics, Dnr M 192-10.

### Animal preparation and monitoring

Thirty-six Swedish-bred (Swedish Landrace) pathogen free pigs with a mean weight of 38 kg (SD ± 4.1, range 32 - 46 kg) were included. The pigs were fasted overnight with free access to water. At the day of the experiment they were pre-medicated with Ketaminol 150 mg/10 kg (Ketamine 100 mg/ml, Intervet, Danderyd, Sweden) and Rompun 20 mg/10 kg intramuscularly (Xylazin 20 mg/ml, Bayer AG, Leverkusen, Germany). After induction of anaesthesia with thiopental 12.5 mg/kg/dose (Pentothal 100 mg/ml Abbott, Stockholm, Sweden) the animals were orally intubated with cuffed endotracheal tubes. To maintain anaesthesia, a slow infusion of (Fentanyl 1.25 μg/ml (Fentanyl Pharmalink AB, Stockholm, Sweden) in buffered glucose (25 mg/ml) was started at a rate of 2 ml/min and adjusted as needed. During the anaesthesia, meprobamat (Mebumal DAK Copenhagen Denmark) and/or thiopental was titrated if needed in small bolus doses. The animals were ventilated with a mixture of nitrous oxide (70%) and oxygen (30%) and normal air using a mechanical Siemens-Elema 900B ventilator (Siemens Elema Solna, Sweden) in the volume-controlled mode, adjusted in order to obtain normoventilation maintaining end tidal carbon dioxide at 4.5 - 5.5 kPa. In addition, the animals were monitored with a three lead electrocardiogram using an IntelliVue MP90 monitoring system (Philips, Eindhoven, The Netherlands). A 6 F FL3.5 diagnostic catheter (Boston Scientific Scimed, Maple Grove, MN, USA) was introduced through the left carotid artery into the ostium of the left main coronary artery. This catheter was used to place a 0.014-inch, 12 MHz pulsed Doppler flow velocity transducer (FloWire® Volcano Inc., San Diego, CA, USA) into the mid-portion of the left anterior descending artery (LAD). A 7.5 F Continuous Cardiac Output Pulmonary Artery Catheter™ (Edwards Lifesciences, Irvine, CA, USA) was inserted through the surgically exposed right jugular vein. Central venous pressure (CVP) was measured in the right atria via a separate transducer. A 6 F pig-tail catheter was placed in the ascending aorta for arterial blood pressure (ABP) measurement. Ten-thousand units of un-fractioned heparin (LEO Pharma AB, Malmoe Sweden) was given intravenously at the start of the catheterisation. The procedures were performed in an experimental catheterization fluoroscopy laboratory (Shimadzu Corp., Kyoto, Japan).

### Experimental protocol

**Figure 1:**
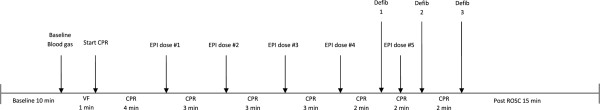
**Experimental time line.** MCC = mechanical chest compressions, VF = ventricular fibrillation, CPR = cardiopulmonary resuscitation, EPI = epinephrine, defib = defibrillation, min = minutes, ROSC = return of spontaneous circulation, Drug administration; either 0.02 mg EPI/kg/dose or 0.03 mg EPI/kg/dose or NaCl , was administrated after 4 minutes of MCC and then every 3^rd^ minute during the 15 minutes of MCC.

A flow chart of the experiment is presented in Figure 1. Pigs were randomized using sealed envelopes. The person opening the envelope prepared the prescribed drug while the remainder of the researchers were blinded to the drug administered during the experiment. The animals received EPI 0.02 mg/kg/dose as previously described in a study by Pytte et al [26] or 0.03 mg/kg/dose as described in a study by Ristagno et al [27] or saline (control). Each syringe was diluted to a total of 10 ml. After preparation and a baseline period, VF was induced using a 9 V direct current (Duracell Battery, Procter & Gamble, Cincinnati OH, USA) between a skin electrode and an intracardiac needle inserted to the epicardium with a stimulation time between 5 – 10 seconds, resulting in an instant loss of ABP and LAD flow. After one minute of untreated VF, MCC and manual ventilation at a rate of 8 – 10 inflations/minute with 100% of oxygen was started and continued for 15 min prior to defibrillation. For standardized chest compressions an electrically driven MCC device was used (LUCAS™2, Physio-Control/Jolife AB, Lund, Sweden). At 5, 8, 11 and 14 minutes after VF-induction an injection of the allocated drug/saline was administered followed by a flush of 10 ml NaCl via a peripheral cannula in a vein in the ear. The rationale behind a 16 minute VF period (1 minute of untreated VF followed by 15 minutes of MCC) was an attempt to reflect a CA situation in the human cardiac catheterisation laboratory with prolonged advanced CPR in connection with PCI [25].

After 15 minutes of VF and MCC, the first defibrillation (LIFEPAK 12 Defibrillator/Monitor, Physio-Control, Redmond, Wa, USA) was attempted. If ROSC was not obtained, a fifth dose of EPI was given and repeated defibrillations were performed as needed for a total of 3 times, with 2-minute intervals during MCC. ROSC was defined as a stable circulation with an ABP > 60 mmHg for 15 minutes after defibrillation. After 15 minutes of ROSC the animals were euthanized with 40 mmol potassium injected into the pulmonary artery catheter.

### Measurements and analysis

Arterial blood pressure and CVP were continuously measured using a sampling rate at 1000 Hz (AD Instruments Inc, Colorado Springs, CO, USA). Hemodynamic parameters were digitally recorded using Chart v4.2 (AD Instruments Inc, Colorado Springs, CO, USA). Coronary perfusion pressure was calculated as the difference between the arterial-end diastolic pressure and the venous right atrial-end diastolic pressure [28]. The maximum peak of CPP (P_max_) was depicted at 20, 60, 120, 180 seconds after initiation of MCC, at the time of every EPI injection and when CPP reached the highest value after each injection. In the control group CPP was depicted 90 seconds after each saline injection. Continuous coronary APV were displayed and recorded using the Doppler flow wire connected to a FloMap monitor (Cardiometrics, Mountain View, CA). The APV was analysed in visual artefact free zones concomitantly with P_max_.

Analog ECG signals were digitized and converted from a time to a frequency domain by fast Fourier transformation at a sampling rate of 250 Hz. Amplitude spectrum area was calculated as the sum of the products of individual frequencies between 8 and 48 Hz at P_max_. The measurements were performed throughout the 16 minutes of VF as median values of every 10 second period. Time to P_max_ were analyzed in the EPI-groups. Survival was defined as stable ROSC for 15 min post successful defibrillation and assessed in each group.

All analyses of these parameters were performed on the three groups and on a merged group including the two EPI-groups (EPI-all).

### Statistics

Analyses were performed comparing data in the control group with those of each EPI dose group and a combination of the 2 EPI-groups. Continuous variables are presented as their mean ± SD or median and 25^th^ to 75^th^ interquartile. Categorical values are presented as numbers and percentages. Fisher’s exact test was used for comparing categorical variables. The Mann-Whitney U test was used for comparing unpaired continuous variables. The Kruskal – Wallis test was used to compare multiple median values when time to peak maximum was compared.

## Results

No difference was seen during the baseline period regarding analysed measurements between any groups (Table 1).

**Table 1:** **Baseline parameters**

	NaCl (n = 12)		EPI all (n = 24)		P-value	EPI 0.02 mg/kg/dose (n = 12)		P-value	EPI 0.03 mg/kg/dose (n = 12)		P-value
**Syst ABP**	152	(137 - 167)	151	(136 - 163)	0.5347	149	(136 - 172)	0.4357	152	(136 - 172)	0.7950
**Diast ABP**	96	(79 - 122)	96	(89 - 106)	0.6996	93	(89 - 99)	0.7075	100	(78 - 107)	0.7950
**Mean ABP**	115	( 99 - 139)	114	(105 - 124)	0.5347	113	(105 - 118)	0.5067	116	(954 - 126)	0.7075
**Max CVP**	12	(10 - 13)	11	(10 -13)	0.6505	11	(10 - 13)	0.5444	11	(10 -13)	0.8852
**Min CVP**	8	(5 - 10)	7	(6 - 8)	0.4502	7	(4 - 8)	0.2855	7	(6 - 9)	0.8399
**Mean CVP**	10	(8 - 11)	9	(8 - 11)	0.7944	9	(8 - 11)	0.7350	9	(8 - 11)	0.9310
**ETCO**_**2**_	4.9	(4.3 - 5.0)	4.5	(4.1 - 4.9)	0.5642	4.8	(4.4 - 5.0)	0.8691	4.3	(4.0 -4.7)	0.2727
**APV**	17	(12 - 28)	17	(12 - 24)	0.7677	20	(13 - 26)	0.8399	15	(11 - 21)	0.4417
**Heart rate**	88	(58 - 97)	75	(64 - 91)	0.7455	77	(71 - 93)	0.8955	71	(56 - 90)	0.4701
**pH**	7.473	(7.420-7.505)	7.484	(7.402-7.510)	0.9331	7.468	7.414-7.529)	0.7950	7.485	(7.3875-7.503)	0.6650
**PCO**_**2**_	5.3	(5.0-6.5)	5.4	(5.1-5.9)	0.7246	5.5	(5.1-5.9)	0.9310	5.2	(4.9-5.9)	0.6236
**PO**_**2**_	27.2	(16.6-37.1)	20.2	(17.2-33.9)	0.6030	23.0	(17.3-33.2)	0.8174	20.2	(16.4-33.9)	0.5254
**ABE**	6.0	(4.6-7.3)	5.9	(2.8-7.8)	0.9065	6.8	(3.2-8.7)	0.7075	5.5	(2.5-6.1)	0.2855

NaCl = Saline, EPI = epinephrine, Syst = Systolic, ABP = arterial blood pressure (mmHg), Diast = Diastolic, Max = maximum.

CVP = central venous pressure (mmHg), Min = minimum, ETCO_2_ = end tidal carbon dioxide (kPa), APV = average peak velocity (cm/s).

Heart rate (beats/minute), PCO_2_ = partial pressure carbon dioxide, (kPa), PO_2_ = partial pressure oxygen (kPa).

ABE = arterial base excess (mmol/l). All values are expressed as median, 25th and 75th interquartile.

### CPP

**Figure 2:**
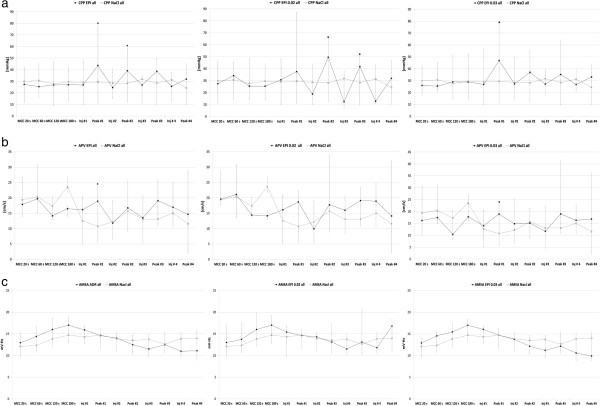
**Peak maximum development of coronary perfusion pressure, coronary artery average peak blood flow velocity and amplitude spectrum area. a** = Development of CPP in the combined group, (0.02 mg EPI/kg/dose and 0.03 mg EPI/kg/dose) compared to control group (NaCl) during the MCC-period (15 min). Significant difference between EPI-group compared to control group, * = p <0.05. **b** = Development of APV in the combined group, (0.02 mg EPI/kg/dose and 0.03 mg EPI/kg/dose) compared to control group (NaCl) during the MCC-period (15 min). Significant difference between EPI-group compared to control group, * = p <0.05. **c** = Development of AMSA in the combined group, (0.02 mg EPI/kg/dose and 0.03 mg EPI/kg/dose) compared to NaCl during the MCC-period (15 min). EPI = epinephrine, NaCl = saline, CPP = coronary perfusion pressure (mmHg), inj = injection number 1- 4, Peak 1 – 4 corresponding to injection 1 - 4, APV = average peak velocity (cm/s), AMSA = amplitude spectrum area (mV · Hz).

During the first 4 minutes of MCC and at the time of the first EPI injection, there were no significant differences in CPP between the control group (n = 12), EPI-all group (n = 24), EPI 0.02 mg/kg/dose (n = 12) or EPI 0.03 mg/kg/dose (n = 12). During the following period of MCC there was a significant increase in CPP at P_max_ after EPI injection 1 and 2 in the EPI-all group (p = 0.022, p = 0.016), compared to the control group but not after injection 3 and 4. We found a significant increase in CPP after injection 2 and 3 in EPI 0.02 mg/kg/dose compared to the control group (p = 0.023, p = 0.027). When comparing EPI 0.03 mg/kg/dose to the control group there was a statistical significant difference at P_max_ following injection 1 (p = 0.013) but not at P_max_ following injection 2, 3 and 4 (Table 2, Figure 2a).

**Table 2:** **Physiological values of CPP during 15 minutes of MCC-time**

	CPP NaCl (n = 12)		CPP EPI all (n = 24)		P-value	CPP EPI 0.02 mg/kg/dose (n = 12)		P-value	CPP EPI 0.03 mg/dose (n = 12)		P-value
**MCC 20 s**	30	(23-42)^a^	27	(12-40)^e^	0.4141	28	(12-46)^b^	0.7818	26	(18-37)^a^	0.2855
**MCC 60 s**	31	(23-36)^a^	25	(16-45)^d^	0.9331	34	(14-46)^a^	0.6650	25	(21-46)^a^	0.7950
**MCC 120 s**	28	(14-36)^a^	27	(9-47)^e^	0.7152	25	(16-49)^b^	0.5588	29	(25-52)^b^	0.6891
**MCC 180 s**	29	(14-39)^a^	27	(13-42)^f^	0.8712	25	(14-44)^b^	1.0000	29	(21-53)^c^	0.9737
**Inj #1**	29	(10-40)^b^	27	(11-49)^n^	0.9368	31	(11-48)^b^	0.9476	27	(14-57)^c^	0.9719
**Peak #1**	29	(11-41)^c^	44	(31-78)^e^	0.0219	38	(28-78)^b^	0.1489	47	(39-77)^a^	0.0134
**Inj #2**	29	(15-41)^b^	25	(16-38)^e^	1.0000	19	(10-44)^a^	0.6009	27	(24-50)^b^	0.5545
**Peak #2**	28	(12-36)^c^	39	(31-59)^e^	0.0160	50	(31-65)^a^	0.0229	37	(29-56)^b^	0.0620
**Inj #3**	32	(18-35)^c^	27	(11-36)^d^	0.9849	12	(7-38)^a^	0.6209	27	(25-39)^a^	0.6682
**Peak #3**	28	(10-38)^c^	39	(29-50)^e^	0.1434	42	(32-50)^a^	0.0265	35	(28-64)^a^	0.1213
**Inj # 4**	31	(21-32)^b^	26	(12-38)^f^	0.9848	13	(11-43)^c^	0.5974	27	(23-39)^a^	0.6891
**Peak #4**	24	10-37)^c^	32	(24-45)^e^	0.1894	32	(22-47)^b^	0.2453	33	(29-44)^a^	0.2766

CPP = Coronary perfusion pressure (mmHg), NaCl = Saline, EPI = epinephrine, MCC = Mechanical chest compressions, Inj = injection, a: n = 12, b: n = 11, c: n = 10, d: n = 24, e: n = 23, f: n = 22, g: n = 21. Peak values at 20 , 60, 120, 180 seconds after start of MCC, at each time point of the 4 drug injections (4, 7, 10, 13 minutes after start of MCC) and the corresponding peak to each injection for NaCl compared to EPI. All values are expressed as median, 25th and 75th interquartile.

### APV

During the first 4 minutes of VF there were no statistically significant differences in APV between any groups. During the following period of MCC there was a significant increase in APV after injection 1when comparing the control group to the EPI-groups except for EPI 0.02 mg/kg/dose group, in which the APV increase was only borderline (p = 0.056). We could not detect any change in APV after the subsequent EPI injections (Table 3, Figure 2b).

**Table 3:** Physiological values of APV during 15 minutes of MCC-time

	APV NaCl (n = 12)		APV EPI all (n = 24)		P-value	APV EPI 0.02 mg/kg/dose (n = 12)		P-value	APV EPI 0.03 mg/kg/EPI (n = 12)		P-value
**MCC 20 s**	19	(12-28)^a^	18	(12-22)^h^	0.3571	20	(13-24)^a^	0.5444	16	(11-32)^b^	0.3401
**MCC 60 s**	20	(15-29)^a^	20	(11-24)^h^	0.4141	21	(12-25)^a^	0.8399	17	(10-22)^b^	0.2071
**MCC 120 s**	17	(12-25)^a^	14	(9-21)^h^	0.3220	14	(10-21)^a^	0.4025	10	(8-21)^b^	0.3401
**MCC 180 s**	24	(17-29)^a^	17	(11-26)^g^	0.0901	14	(10-30)^a^	0.0606	18	(12-24)^b^	0.1316
**Inj #1**	13	(12-20)^c^	16	(8-20)^m^	0.8536	16	(9-20)^e^	0.9646	14	(7-25)^e^	0.6893
**Peak #1**	11	(9-15)^c^	19	(15-26)^j^	0.0109	19	(12-23)^e^	0.0561	19	(15-42)^b^	0.0183
**Inj #2**	12	(8-18)^e^	12	(9-20)^i^	0.8262	10	(8-16)^b^	0.5915	15	(12-21)^b^	0.2303
**Peak #2**	16	(6-19)^e^	17	(13-31)^l^	0.1899	18	(13-36)^d^	0.1629	15	(13-35)^e^	0.4309
**Inj #3**	13	(11-20)^d^	14	(7-22)^k^	0.5892	16	(9-26)^c^	0.9674	12	(7-18)^e^	0.3606
**Peak #3**	13	(9-22)^b^	19	(14-31)^j^	0.5892	19	(15-29)^a^	0.1661	19	(14-33)^f^	0.3191
**Inj # 4**	15	(9-20)^b^	17	(9-21)^j^	0.9314	19	(8-29)^d^	0.8792	16	(10-21)^c^	1.0
**Peak #4**	12	(8-21)^b^	15	(10-29)^m^	0.6749	14	(12-22)^e^	0.6497	17	(8-37)^e^	0.8365

APV = Average peak velocity (cm/s), NaCl = Saline, EPI = epinephrine, MCC = mechanical chest compressions, Inj = injection, a: n = 12, b: n = 11, c: n = 10, d: n = 9, e: = 8, f: n = 7, g: n = 24, h: n = 23, i: n = 21, j: n = 19, k: n = 18, l: n = 17, m: n = 16. Peak values at 20 , 60, 120, 180 seconds after start of MCC, at each time point of the 4 drug injections (4, 7, 10, 13 minutes after start of MCC) and the corresponding peak to each injection for NaCl compared to EPI. All values are expressed as median, 25th and 75th interquartile.

### AMSA

There were no statistical differences in AMSA, when comparing the control group to any of the EPI-groups. (Table 4, Figure 2c).

**Table 4:** Physiological values of AMSA during 15 minutes of MCC-time

	AMSA NaCl		AMSA EPI All		P-value	AMSA EPI 0.02 mg/kg/dose		P-value	AMSA EPI 0.03 mg/kg/dose		P-value
**MCC 20 s**	12.1	(9.6-14.0)^b^	13.0	(10.9-16.0)^d^	0.4099	13.0	(11.8-13.0)^c^	0.3913	13.1	(10.6-15.0)^a^	0.5752
**MCC 60 s**	12.4	(10.8-15.3)^b^	14.4	(11.4-16.8)^d^	0.4990	13.7	(10.8-17.6)^c^	0.7751	14.6	(13.0-15.8)^a^	0.4288
**MCC 120 s**	13.8	(12.1-16.3)^b^	16.0	(14.2-18.7)^d^	0.1974	16.0	(14.2-19.8)^c^	0.1779	15.5	(13.8-17.6)^a^	0.3734
**MCC 180 s**	14.7	(13.3-17.9)^b^	17.0	(12.8-19.0)^d^	0.3749	17.0	(12.8-19.3)^c^	0.4877	17.0	(15.3-18.3)^a^	0.4288
**Inj #1**	14.3	(12.7-16.8)^b^	15.9	(13.9-16.7)^d^	0.4725	15.4	(9.7-16.7)^c^	0.4142	16.1	(13.3-16.7)^a^	0.6682
**Peak #1**	14.7	(11.5-15.9)^b^	14.6	(13.0-16.2)^d^	0.6121	14.6	(13.8-15.9)^c^	0.7133	14.7	(12.7-16.2)^a^	0.6444
**Inj #2**	13.9	(11.0-15.4)^b^	14.0	(11.6-14.5)^d^	0.8824	14.3	(13.0-14.9)^c^	0.7751	13.8	(11.6-14.3)^a^	0.6209
**Peak #2**	13.5	(10.4-14.7)^b^	12.5	(11.0-14.6)^d^	1.0000	13.1	(11.9-17.5)^c^	0.5956	12.2	(10.4-13.9)^a^	0.6682
**Inj #3**	13.7	(11.4-14.8)^b^	11.5	(9.7-13.8)^d^	0.2285	11.5	(10.2-20.9)^c^	0.5676	11.2	(9.5-11.7)^a^	0.1616
**Peak #3**	12.7	(10.6-15.5)^b^	12.5	(11.1-14.5)^d^	0.9831	13.1	(10.2-20.9)^c^	0.5954	12.2	(11.3-14.3)^a^	0.7169
**Inj # 4**	13.9	(11.3-15.3)^b^	11.0	(9.7-13.5)^d^	0.1696	11.8	(10.5-14.3)^c^	0.6534	10.6	(8.8-12.2)^a^	0.0806
**Peak #4**	14.0	(11.4-15.3)^e^	11.0	(8.9-15.9)^d^	0.8841	16.8	(9.9-11.1)^c^	0.3165	9.9	(8.8-14.3)^a^	0.2814

AMSA = amplitude spectral area (mV · Hz), NaCl = saline, EPI = epinephrine, MCC = Mechanical chest compressions, Inj = injection, a: n = 12, b: n = 10, c: n = 9, d: n = 21, e: n = 6. Peak values at 20 , 60, 120, 180 seconds after start of MCC, at each time point of the 4 drug injections (4, 7, 10, 13 minutes after start of MCC) and the corresponding peak to each injection for NaCl compared to EPI. All values are expressed as median, 25th and 75th interquartile.

### Time to maximum peak of CPP

The median time to P_max_ following the EPI injections was 50 (interquartile range 48 to 52) seconds (Table 5). There were no significant difference in time to P_max_ between the EPI-groups following injection 1 to 4 (Table 5).

**Table 5:** **Time to peak**

	Inj #1		Inj #2		Inj #3		Inj #4		P-value
**Epinephrine all**	51	(32-66)^c^	53	(41-58)^c^	49	(41-70)^c^	56	(44-78)^c^	0.392
**Epinephrine 0.02 mg/kg/dose**	48	(39-49)^b^	48	(40-52)^a^	52	(50-60)^a^	47	(43-59)^b^	0.440
**Epinephrine 0.03 mg/kg/dose**	48	(38-60)^a^	51	(40-56)^b^	51	(46-62)^b^	49	(44-65)^a^	0.440

Time duration from drug administration to peak time (seconds) /P_max_ of CPP for the 4 different drug injections. Inj = Injection, mg = milligram, kg = kilogram, a; n = 12, b; n = 11, c; n = 23. All Values are expressed as median, 25th and 75th interquartile.

### ROSC

Return of spontaneous circulation was achieved in 10/12 (84%) animals in the control group compared with 7/12 (58%) in each EPI-group (p = 0.37).

## Discussion

This study shows that intravenous administration of recommended doses of EPI during prolonged advanced CPR with MCC increases the perfusion pressure in the coronary vessel as expected. However, the effect of EPI seems to be weakened after three out of four injections when compared to controls. We also found, that the increase in CPP only transfers into an increase in APV after the first injection of EPI and no effect could be detected in AMSA. Human studies have shown that administration of EPI according to guidelines recommendations has a positive effect to attain ROSC but a worse neurological/survival outcome at discharge from hospital [8, 29]. This has also been observed when using higher cumulative doses of EPI [4]. In the present study, numerically fewer animals receiving EPI obtained ROSC compared to the control group, although the difference was not significant. Larger studies are needed to demonstrate a possible negative effect on survival of repeated intravenous EPI-doses during CPR.

In this study median time to P_max_ was 50 (48 – 52) seconds. Pytte et al showed a median time to peak after 53 seconds with MCC with a CC-depth at 45 mm and after 83 seconds with manual CC according to guidelines from 2005 [26]. Thus both studies support a better circulation in terms of time to peak of CPP created by MCC.

In addition to an adequate CPP, myocardial perfusion is dependent on coronary artery flow velocity. In a previous study it was shown that APV was highly correlated to CPP during prolonged CPR with MCC [20], a study that was conducted without administration of EPI. In another study Mayr et al demonstrated that repeated injections of vasopressin resulted in an increased coronary blood flow in pigs with induced VF circulated with a cardio-pulmonary bypass technique in a low flow state [30]. Brown et al showed that high doses of EPI elevated myocardial blood flow to a higher extent compared with standard doses [24]. In our study we could only detect a significant rise in APV after the first EPI injection. Thus, the increase in CPP after EPI-injection 2 and 3 was not accompanied by a concomitant elevation in APV indicating a rise in local vascular resistance and a lesser amount of oxygenated blood reaching the myocardium, corresponding to the findings of Brown et al using standard doses of EPI. The different circulation techniques and ventilation rates used in these studies may have significant impact on differences in their results. Still, the importance to keep CPP at a high level may be questioned, since the elevated pressure values caused by EPI in 3 out of 4 injections was not accompanied by an increase in APV.

Amplitude spectrum area has been associated with a high positive and negative predictive value to obtain a successful defibrillation in both experimental and human studies [31, 32]. In one study the analyses were performed only on data from defibrillators used in CA-cases, no other information about CPR-time or administered EPI during the resuscitation were recorded [32], rendering further comparisons difficult to our experimental study.

Furthermore, human and experimental studies have shown that a CPP above 15 mmHg markedly increases the possibility to attain ROSC following defibrillation [9, 10]. Hence, to strive towards a CPP above 15 mmHg during CPR in order to attain ROSC seems logical. Several experimental studies have shown that an EPI-induced elevation of CPP leads to a higher probability of subsequent ROSC [11, 12, 33]. It is important to stress, that in these studies only one injection of EPI was administered.

In studies investigating repeated injections of EPI, Bar-Joseph et al showed a significant increase in CPP only after the first injection of repeated doses of high dose EPI (0.1 mg/kg) [22], and Cairns et al showed a significant increase in CPP only in the animals who attained ROSC after first EPI injection followed by defibrillation [21]. We found a significant increase of CPP in 3 out of 4 injections, however less pronounced after injection 2 and 3. The studies by Bar-Joseph et al and Cairns et al used longer periods of untreated VF-period simulating OHCA, higher doses of EPI and defibrillation attempts after each injection of EPI [21, 22]. Our goal was to simulate a frequently occurring situation in the coronary catheterisation laboratory, with a short period of untreated VF, followed by repeated administrations of EPI in conjunction with defibrillation and resistant VF. As opposed to the previous studies we administrated doses of EPI which were closer to those recommended in the guidelines. In addition, the different periods of untreated VF time in, previous experimental settings may influence the metabolic status and consumption of endogenously produced EPI in the animals at the initiation of CPR, which in turn may affect the response to EPI. We also used slightly different CC-techniques and ventilation rates [21, 22], which also may affect the distribution and effect of administered drugs.

In regards to cardiac pressure and their influence on the bioelectrical activity during VF a high CPP correlates to a high AMSA in some studies [13, 16]. Similar to our study, Achleitner et al showed an elevated mean fibrillation frequency and VF mean amplitude, during basic life support [23]. On the contrary, AMSA showed an insignificant tendency to decline in the EPI-groups in our study despite an increase in CPP, similar to previous findings [23]. Despite different CPR models and EPI dosages, the results in AMSA were similar in previous study [23] compared to current study. In the present study EPI was unable to induce a rise in AMSA, despite increased CPP and therefore it contributed to a successful defibrillation in only 7 out of 12 animals in each EPI-group. A possible explanation for this result may be an increased resistance resulting in a decreased myocardial microcirculatory blood flow induced by EPI, which has also been described in capillaries of the brain [34]. The lack of effects on AMSA following the repeated doses of EPI may accordingly be a result of a successively diminished myocardial tissue perfusion caused by EPI throughout the experiment. Thus, the initial injections of EPI may serve a purpose, but repeated injections may not be beneficial and may in fact be detrimental in subjects with VF who are reasonably circulated with manual CC or MCC. Further research is needed to determine the optimal dosages and frequency of EPI-injections during CPR.

### Limitations

The experimental set up primarily reflects a very specific in-hospital CA scenario including a prompt response to a CA with CC followed by a prolonged CPR situation, for instance in the catheterisation laboratory with a therapy resistant VF and MCC. Since the animals were young, healthy and without coronary artery disease, it may be difficult to extrapolate our findings into an unselected patient category suffering CA. The study was not powered to evaluate differences in survival. EPI was administered according to guidelines, and the results should be interpreted accordingly. Different regimens of dosage and time intervals might give different results.

## Conclusion

We conclude that repeated intravenous injections of EPI administered according to resuscitation guidelines during CA, increases APV after the first injection only despite an increase in CPP after 3 out of 4 EPI injections. We found no difference in AMSA values at any measuring point.

## References

[1] Redding JS, Pearson JW (1962). Resuscitation from asphyxia. JAMA.

[2] Nolan JP, Soar J, Zideman DA, Biarent D, Bossaert LL, Deakin C, Koster RW, Wyllie J, Bottiger B (2010). European Resuscitation Council Guidelines for Resuscitation 2010 Section 1. Executive summary. Resuscitation.

[3] Neumar RW, Otto CW, Link MS, Kronick SL, Shuster M, Callaway CW, Kudenchuk PJ, Ornato JP, McNally B, Silvers SM, Passman RS, White RD, Hess EP, Tang W, Davis D, Sinz E, Morrison LJ (2010). Part 8: adult advanced cardiovascular life support: 2010 American Heart Association Guidelines for Cardiopulmonary Resuscitation and Emergency Cardiovascular Care. Circulation.

[4] Behringer W, Kittler H, Sterz F, Domanovits H, Schoerkhuber W, Holzer M, Mullner M, Laggner AN (1998). Cumulative epinephrine dose during cardiopulmonary resuscitation and neurologic outcome. Ann Intern Med.

[5] Gueugniaud PY, Mols P, Goldstein P, Pham E, Dubien PY, Deweerdt C, Vergnion M, Petit P, Carli P (1998). A comparison of repeated high doses and repeated standard doses of epinephrine for cardiac arrest outside the hospital. European Epinephrine Study Group. N Engl J Med.

[6] Mukoyama T, Kinoshita K, Nagao K, Tanjoh K (2009). Reduced effectiveness of vasopressin in repeated doses for patients undergoing prolonged cardiopulmonary resuscitation. Resuscitation.

[7] Olasveengen TM, Sunde K, Brunborg C, Thowsen J, Steen PA, Wik L (2009). Intravenous drug administration during out-of-hospital cardiac arrest: a randomized trial. JAMA.

[8] Jacobs IG, Finn JC, Jelinek GA, Oxer HF, Thompson PL (2011). Effect of adrenaline on survival in out-of-hospital cardiac arrest: A randomised double-blind placebo-controlled trial. Resuscitation.

[9] Paradis NA, Martin GB, Rivers EP, Goetting MG, Appleton TJ, Feingold M, Nowak RM (1990). Coronary perfusion pressure and the return of spontaneous circulation in human cardiopulmonary resuscitation. JAMA.

[10] Niemann JT, Criley JM, Rosborough JP, Niskanen RA, Alferness C (1985). Predictive indices of successful cardiac resuscitation after prolonged arrest and experimental cardiopulmonary resuscitation. Ann Emerg Med.

[11] Kern KB, Ewy GA, Voorhees WD, Babbs CF, Tacker WA (1988). Myocardial perfusion pressure: a predictor of 24-hour survival during prolonged cardiac arrest in dogs. Resuscitation.

[12] Reynolds JC, Salcido DD, Menegazzi JJ (2010). Coronary perfusion pressure and return of spontaneous circulation after prolonged cardiac arrest. Prehosp Emerg Care.

[13] Marn-Pernat A, Weil MH, Tang W, Pernat A, Bisera J (2001). Optimizing timing of ventricular defibrillation. Crit Care Med.

[14] Noc M, Weil MH, Gazmuri RJ, Sun S, Biscera J, Tang W (1994). Ventricular fibrillation voltage as a monitor of the effectiveness of cardiopulmonary resuscitation. J Lab Clin Med.

[15] Povoas HP, Bisera J (2000). Electrocardiographic waveform analysis for predicting the success of defibrillation. Crit Care Med.

[16] Reynolds JC, Salcido DD, Menegazzi JJ (2012). Correlation between coronary perfusion pressure and quantitative ECG waveform measures during resuscitation of prolonged ventricular fibrillation. Resuscitation.

[17] Segal J (1993). Applications of coronary flow velocity during angioplasty and other coronary interventional procedures. Am J Cardiol.

[18] Ofili EO, Labovitz AJ, Kern MJ (1993). Coronary flow velocity dynamics in normal and diseased arteries. Am J Cardiol.

[19] Ninomiya Y, Hamasaki S, Saihara K, Ishida S, Kataoka T, Ogawa M, Orihara K, Oketani N, Fukudome T, Okui H, Ichiki T, Shinsato T, Kubozono T, Mizoguchi E, Ichiki H, Tei C (2008). Comparison of effect between nitrates and calcium channel antagonist on vascular function in patients with normal or mildly diseased coronary arteries. Heart Vessels.

[20] Wagner H, Madsen Hardig B, Steen S, Sjoberg T, Harnek J, Olivecrona GK (2011). Evaluation of coronary blood flow velocity during cardiac arrest with circulation maintained through mechanical chest compressions in a porcine model. BMC Cardiovasc Disord.

[21] Cairns CB, Niemann JT (1998). Hemodynamic effects of repeated doses of epinephrine after prolonged cardiac arrest and CPR: preliminary observations in an animal model. Resuscitation.

[22] Bar-Joseph G, Weinberger T, Ben-Haim S (2000). Response to repeated equal doses of epinephrine during cardiopulmonary resuscitation in dogs. Ann Emerg Med.

[23] Achleitner U, Wenzel V, Strohmenger HU, Krismer AC, Lurie KG, Lindner KH, Amann A (2000). The effects of repeated doses of vasopressin or epinephrine on ventricular fibrillation in a porcine model of prolonged cardiopulmonary resuscitation. Anesth Analg.

[24] Brown CG, Werman HA, Davis EA, Hobson J, Hamlin RL (1987). The effects of graded doses of epinephrine on regional myocardial blood flow during cardiopulmonary resuscitation in swine. Circulation.

[25] Wagner H, Terkelsen CJ, Friberg H, Harnek J, Kern K, Lassen JF, Olivecrona GK (2010). Cardiac arrest in the catheterisation laboratory: A 5-year experience of using mechanical chest compressions to facilitate PCI during prolonged resuscitation efforts. Resuscitation.

[26] Pytte M, Kramer-Johansen J, Eilevstjonn J, Eriksen M, Stromme TA, Godang K, Wik L, Steen PA, Sunde K (2006). Haemodynamic effects of adrenaline (epinephrine) depend on chest compression quality during cardiopulmonary resuscitation in pigs. Resuscitation.

[27] Ristagno G, Tang W, Huang L, Fymat A, Chang YT, Sun S, Castillo C, Weil MH (2009). Epinephrine reduces cerebral perfusion during cardiopulmonary resuscitation. Crit Care Med.

[28] Otlewski MP, Geddes LA, Pargett M, Babbs CF (2009). Methods for calculating coronary perfusion pressure during CPR. Cardiovasc Eng.

[29] Olasveengen TM, Wik L, Sunde K, Steen PA (2012). Outcome when adrenaline (epinephrine) was actually given vs. not given - post hoc analysis of a randomized clinical trial. Resuscitation.

[30] Mayr VD, Wenzel V, Muller T, Antretter H, Rheinberger K, Lindner KH, Strohmenger HU (2004). Effects of vasopressin on left anterior descending coronary artery blood flow during extremely low cardiac output. Resuscitation.

[31] Ristagno G, Gullo A, Berlot G, Lucangelo U, Geheb E, Bisera J (2008). Prediction of successful defibrillation in human victims of out-of-hospital cardiac arrest: a retrospective electrocardiographic analysis. Anaesth Intensive Care.

[32] Ristagno G, Li Y, Fumagalli F, Finzi A, Quan W (2013). Amplitude spectrum area to guide resuscitation-a retrospective analysis during out-of-hospital cardiopulmonary resuscitation in 609 patients with ventricular fibrillation cardiac arrest. Resuscitation.

[33] Lindberg L, Liao Q, Steen S (2000). The effects of epinephrine/norepinephrine on end-tidal carbon dioxide concentration, coronary perfusion pressure and pulmonary arterial blood flow during cardiopulmonary resuscitation. Resuscitation.

[34] Ristagno G, Sun S, Tang W, Castillo C, Weil MH (2007). Effects of epinephrine and vasopressin on cerebral microcirculatory flows during and after cardiopulmonary resuscitation. Crit Care Med.

[CR35] The pre-publication history for this paper can be accessed here:http://www.biomedcentral.com/1471-2261/14/199/prepub

